# Memoir on Jalap

**Published:** 1809-05

**Authors:** M. Desfontaines


### *Memoir on Jalap, by* M. Desfontaines.

JALAP grows naturally in Mexico, in the environs of the town of Xalapa,* from which it derives its name, and where it is also cultivated in great abundance for commercial speculations. Thierry de Menonville, a distinguished botanist, informs us, in the account published of his journey to Guaxaca, printed in 1787, that jalap is also very common in the country around Vera Cruz, but that the inhabitants were ignorant of this circumstance, until it was pointed out to them, after having been long in the habit of purchasing it from Xalapa, at 3 reals per pound.

The root, the only part of the plant made use of, is very thick and bulbous; it is of an oval or pear-like form, and when fresh, it is white and milky in its interior. From the lower part several roots issue of unequal size, which descend perpendicularly into the ground, and on the surface of the principal root we may remark some depressions, from which the shoots and radicles issue. M. Thierry says, that he has seen roots which weighed 12, 15, and even 25 pounds. They are cut into transverse sections, in order to dry them, and sometimes when they are of a small size, they are cut into halves throughout their whole length, so as to resemble half a pear. They shrivel on being dried, assume a brown colour externally, are grey internally, and they generally contain a resinous oil.

According to Raynal, Europe consumes annually 7,500 quintals, for which 972,000 francs are paid. The best kind is brown, compact, heavy, resinous, and difficult to break: by itself jalap is almost tasteless and inodorous; it acquires a sharpness, however, after being infused some time. When burnt, it diffuses a strong and disagreeable smell.

This drug was unknown to the Greeks and Arabs, and we are indebted for it to the discovery of the new world. The inhabitants of Mexico use great quantities of it as a medicine: from the Mexicans the Europeans acquired a knowledge of its properties; and about the commencement of the 17th century, according to Gaspar Bauhin, it became a staple article of European commerce. *Ante annos duodecim allata est à Massiliensibus Jalapium vel Gelapio vocitatur.*

---

* The Spaniards pronounce the X like the French Ch, which is easily sharpened into the English J.

*vocitatur.* C. Bauh. Pinax. p. 298. He says, that this purgative is preferable to the white Mechoacana, *(convolvulus mechoacana, Linn.)* because it is fitter for evacuating the aqueous humours without griping, and that it fortifies the stomach. According to this author, it may be taken in substance in the dose of a drachm, or rather mixed with syrup of succory, broth, wine, or any other convenient vehicle.

Simon Pauli and Hoffman, confine the dose to 24 grains, and Geoffroy says, that it purges effectually from 12 grains to a scruple; he cautions us however against using jalap in acute fevers, and says, it is at variance with dry and hot temperaments. It is according to him an excellent remedy in dropsies, and, in a word, on all occasions, when we wish to purge off serosities and glary matters; an opinion which is confirmed by the experience of a great many celebrated physicians. Some have advised the weakening of the action of the jalap by mixing it with acids, alkaline salts, oils, or aromatics, such as cinnamon, ginger, &c. But Geoffroy regards these methods as improper, in so far as they change the nature of an excellent purgative, and which is not noxious in itself, when we give it in a small dose; and in the cases that require it, he assures us, that it is always better to take it in substance than in mixture. Another advantage of this remedy is, that being of a low price, the poor may be amply supplied with it. It should seem that the virtues of jalap reside chiefly in the resin it contains; and as it is in more or less abundance, its action varies in the same proportion. This is an inconvenience difficult to avoid, and which it is not easy to remedy, but with which we may equally reproach other medicaments in common use, and the virtues of which are analogous to those of jalap. The resin extracted by means of spirit of wine is a very strong purgative, frequently produces violent pains in the bowels, and for this reason it is dangerous to give it alone. Wedel says, that jalap may be safely administered to infants. Van Swieten advises it to be reduced to powder, bruised with sugar, and mixed with an aromatic in a small quantity. When prepared in this manner, we may take it in wine or water, and it purges efficaciously, and without pain. It is also an excellent remedy against worms and tæniæ. Wepfer mentions several cures made by means of jalap. The resinous and gummy substance it contains is not always in the same quantity, nor in the same proportion as already mentioned, and as has been proved by the various chemical analyses made of this substance. Jalap is also used in veterinary medicine. Miller says, that the brewers and distillers of London employ it in order to hasten fermentation, and that they consume great quantities of it in this way.

The plant which furnishes jalap has been long unknown. Plumier, Tournefort, Geoffroy, and Linnæus, in the first edition of his Materia Medica, thought it to be the garden Nightshade; and for this reason, Tournefort gave it the name of *Jalapa,* and Linnæus that of *Mirabilis Jalapa,* which it still bears.

This author having afterwards remarked a strong resemblance between the bark, the interior texture, and the size of the root of the long flowered nightshade, *Mirabilis Longiflora, Lin.* and that of jalap, thought they might be the same. Vide Amœnit. Acad. tom. 7, p. 308.

Finally, Bergius, after being convinced by experience, that the root of the dichotomous nightshade, *Mirabilis dichotoma,* purged very well, while that of the two preceding kinds had almost no virtue at all, asserted that the dichotomous nightshade was the true jalap, and the editors of the Swedish Pharmacopœia adopted his opinion. But if these authors had an opportunity of comparing the fresh and entire roots of jalap with those of the three kinds of nightshade mentioned above, they would not have fallen into such errors, because they would have presented to them extremely remarkable differences.

Ray, Houstoun, Sloane, and Miller, have nevertheless asserted, that jalap is a species of bind weed; and Linnæus, in his *Mantissa,* published at the end of the *Systema Naturæ,* adopted this opinion. In the second edition of his *Materia Medica,* we also find jalap among the bind weeds.

It is designated in these two works *Convolvulus Jalapa.* Houstoun, who had travelled into that part of Spanish America where jalap grows spontaneously, observed it there, and brought some fresh roots to Jamaica, with a view to their cultivation, but they perished in consequence of the neglect of the person to whom he entrusted their culture. Houstoun, upon his return to England, shewed specimens of the plant dried with its flowers, to Bernard de Jussieu, who was then in London, and this celebrated botanist ascertained that it was a species of bind weed.

Miller, having received some seeds into the garden at Chelsea, they sprang up, and produced very large roots, with vine looking herbaceous stalks, about nine or ten feet high; they were furnished with oval leaves, either entire entire, or lobellated, but none of them flourished. Miller adds, that Houstoun presented him with a drawing, representing the jalap when in flower; and he assures us that it was a bind weed. He also says, that the seeds are furnished with down, a character which distinguishes jalap, as may be seen from the description about to follow.

Murray, in his Materia Medica, has adopted the opinion of Ray, Houstoun, and Miller. Thierry de Menonville, who had been at Xalapa and Vera Cruz, had also observed the jalap plant at both places. This botanist has fully confirmed the sentiments of these authors. We may be convinced by the description drawn up on the spot, and sent to M. Jussieu, who communicated it to me, not only that this plant belongs to the genera of bind weeds, but that the plants cultivated for some years in the parterres of the Museum of Natural History, present no sensible difference, and consequently that it is the true jalap which we possess.

Finally, Woodville, in his work called Medical Botany, has published some observations upon jalap, with a drawing, which furnishes no idea of the organs of fructification.

As the jalap plant has been long known among the bind weed genera, we shall leave it there, although from its simple stigmata it appears rather to belong to the *Ipomœa*. In short, these two genera are established upon characters so little different, that it would perhaps be best to bring them into one genus.

*Convolvulus Jalapa, convolvulus caule volubili, tuberculoso ; foliis cordato ovatis, subrugosis, subtus villosis, integris aut lobatis, pedunculis uni vel multifloris ; filamentis basi tomentosis ; semine lavigero.*

*Bryonia Mechoacana nigricans*. C. B. Pin. 298. Prodr. 135. I. B. Hist. 2, p. 151. *Mechoana nigricans*, Park. Theat. 180. Convolvulus Americanus Jalapium dictus. Ray. Hist. 724.

*Convolvulus radice tuberoso cathartico*, Houstoun, MS. ex Millero. *Convolvulus Jalapa, foliis variis, pedunculis unifloris radice tuberoso*, Miller, Dict. 2edit. 8vo. No. 31.

*Convolvulus caule volubili* ; foliis difformibus, *cordatis angulatis, oblongis lanceolatisque ; pedunculis unifloris*, Lin. Mantissa, 4, p. 43. Syst. veget. 169. Mat. Med. edit. 2, p. 66. Murray, Mat. Med. 1, p. 754. Lamarck Dict. 3. p. 542. *Illustr*. No. 2012. Tab. 104. f. 2. *Convolvulus caule volubili foliis ovatis, subcordatis obtusis , obsolete repandis, subtus villosis, pedunculis unifloris*. Hort. Kew. 1, p. 211. Wild Spec. 2, p. 860. *Woodville*, Med. Bot. No. 5, p. 59.

Root

Root pearshaped, fleshy, milky, very large, externally strewed with depressions not very deep, divided below into several unequal and perpendicular radicles.

From its upper part issue several viny, striated, herbaceous stalks, reddish in young plants, a size larger than a writing quill, divided into long and flexible branches, hairy at their upper extremity, and commonly strewed with small tubercles. They twist around such objects as are near them like ivy, and rise to the height of six or seven metres.

Leaves alternate, oval, or heart-formed, hairy underneath, a little undulated, obtuse, sometimes acute, terminated by a small gland, entire or divided into two, three, or five lobes. Nerves oblique, salient underneath, forming furrows above. Petioli cylindrical, tuberculous, with a longitudinal furrow.

Pedunculi axillary, solitary, pubescent, four or five centimetres long, terminated by one, two, or more flowers; furnished at their upper part with two opposite tubercles, whence issue two small oval bracteæ, which speedily wither and fall off.

Calix permanent, pale green, pubescent, elongated oval, two centimetres long. Five deep divisions, oval, obtuse, convex externally, nearly of equal length, serrated against the tube of the corolla; two are external, the three internal ones are rounded at the summit.

Corolla large, covered with very short and fine down. Tube cylindrical, violet internally, of a pale lilac externally, nine centimetres long. Limbus opened, bell shaped, white or shaded with violet, with five round lobes, not deep, marked with five longitudinal bands, which proceed by shrivelling up from the base to the point, as is the case with all plants of this description; they are striated throughout their whole length, and veined with violet coloured lines. Diameter of the limbus of the corolla equal to the length of the tube.

Five unequal stamina joined together in the centre of the flower; the longest do not project beyond the tube. Filaments cylindrical sharp, white, gradually thinner from bottom to top, furnished below with small violet down, similar to very fine cotton, attached a little above the base of the tube. Antheræ vertical, sagittal, adhering to the filaments by the base; pollen white.

Style filiform, white, of the length of the stamina, surmounted by a thick stigma, depressed, bilobed, strewed with small tubercles. Ovarium oval, sharp.

Capsule

Capsule glittering, oval, thick, brittle, of the size of a filbert, covered by the calix opening into four valves, divided into three or four boxes, each containing one or two seeds, black, oblong, convex outwardly, almost triangular internally with a cicatrix near one of the extremities. The whole of their external surface is covered with fine long down of a reddish colour.

The flowers expand about six or seven o'clock, and shut about eleven o'clock in the morning.

When we compared the above description with that of Thierry de Menonville, we were fully convinced that the jalap cultivated in our Museum, was precisely the same with that of Xalapa, and of Vera Cruz. M. Michaux has also discovered it in a district situated to the southward of Florida, where it grows naturally, besides several other Mexican plants. M. Michaux transplanted it to the Botanical Garden at Charlestown, where he cultivated it with much success. M. Bosc, upon his return from the United States of America, gave some seeds to M. Thouin, and it is to his care that we are indebted for this valuable plant. Thierry says, that jalap delights in arid and sandy grounds, and that it grows in these situations without culture. In the climate of Paris, he admits that it should be brought up in the hot-house, but it is extremely probable that it would succeed in the open air in the south of France, where the temperature resembles that of Florida and Carolina.

We shall conclude this Memoir with observing, that the genus of Bell flowers, besides jalap, contains several other purgative species very much in use; such as scammony, *convolvulus scammonia*, Lin: turbith or turpet C. *turpetum* Lin. le Mechoacan, C. *Mechoacana*, Lin. Sea soldanella, C. *soldanella*, Lin. Hedge Bell-flower C. *sepium*, Lin.

Analogy inclines us to think, that this numerous genus includes many others which have the same property, and that several more ought also to be found among the Ipomæa, because they have the greatest affinity with the bell-flowers. It may not be useless however to remark, that potatoes, which are mild, saccharine, and nutritive, belong to the same series with the purgative plants in question, and that if the virtues of plants generally follow the law of natural relations, this law is not always without exceptions. It would be easy to adduce several other examples.

Addition

*Addition to the above Memoir on Jalap, by M. Michaux.*

M. Desfontaines has observed, that the plants cultivated in the garden of the Museum, came from seeds obtained in the Botanical Garden at Charlestown, which my late father originated, and in which he deposited the vegetables he had collected in various parts of South America.

It was in the last journey he made, however, (in 1778) and in which I accompanied him, that he found jalap for the first time. He subsequently observed it in Georgia and Carolina.

Some years afterwards, the establishment of the Charlestown Garden having been put an end to by order of the American Government, the French minister sent me to Carolina, with instructions to send to France seeds and young plants of all the forest trees which it might be advantageous to naturalise in France, and particularly the *quercus virens,* which grows upon the sea shore in dry sands, and the wood of which is superior to every other kind of oak. I was also instructed to send all the plants which might still be preserved in the Charles-town Garden, after being abandoned for four years. Here I found a very large sized root of jalap, from which several stalks issued, and I collected a considerable quantity of seeds, which I sent to the Board of Agriculture in France. At this season the frosts had already destroyed the stalks of most of the herbaceous plants, and particularly those of the jalap in question. I observed that one third of the root was above ground, and I was astonished to find that it was not injured by the cold. I was told that it had remained uncovered four years, and that it had suffered no injury, although Reaumur's thermometer had been frequently four or five degrees below the freezing point. This observation is worthy of attention, because it confirms the opinion of M. Desfontaines and myself, that jalap may be cultivated in the open air, in the south of France. The large root I have mentioned, grew in a light sandy soil, which seems to agree with it best. Not knowing if the seeds sent to the museum had succeeded, I determined to convey it to France, notwithstanding its enormous size. I packed it carefully in fresh moss, and although it was four months on the voyage, it arrived in good condition at the garden of the museum at Paris, where it produced some new plants.

Messrs. Desfontaines and Thouin weighed it before putting it into the earth, its weight was 47 pounds 3 ounces; and certainly exceeded 50 pounds weight when it was originally pulled up in America. I was under the necessity of cutting off several branches, from their being too long to be admitted into the box intended for it: some of them were upwards of an inch in diameter.

This root has long continued in full vegetation, and has sent out a great number of vigorous stalks.

